# Randomisierte Phase-II-Studie zur an der PET-Response orientierten multimodalen Therapie des Speiseröhrenkrebses – Ergebnisse des CALGB 80803 (Alliance) Trial

**DOI:** 10.1007/s00066-021-01898-8

**Published:** 2022-01-11

**Authors:** Lukas Bauer, Kristin Lang

**Affiliations:** Universitätsklinikum Heidelberg, Im Neuenheimer Feld 400, 69120 Heidelberg, Deutschland

## Ziel/Hintergrund der Arbeit

Bewertung des Nutzens einer frühzeitigen Beurteilung des Ansprechens auf Chemotherapie durch eine Positronenemissionstomographie (PET), um die Therapie bei Patienten mit Adenokarzinomen des Ösophagus oder des ösophagogastralen Übergangs anzupassen.

## Methode/Patientengut

Nach einer Baseline-PET wurden die Patienten nach dem Zufallsprinzip einer Induktionschemotherapie zugeteilt: modifiziertes Oxaliplatin, Leucovorin und Fluorouracil (FOLFOX) oder Carboplatin plus Paclitaxel (CP). Nach der Induktionschemotherapie wurde die PET wiederholt und die Veränderung des maximal standardisierten „uptake“ (SUV) gegenüber dem Ausgangswert bewertet. PET-Nonresponder (< 35 % Abnahme des SUV) wechselten während der Radiochemotherapie (50,4 Gy in 28 Fraktionen, RCT) zu einer anderen Chemotherapie. PET-Responder (≥ 35 % Abnahme des SUV) wurden mit der gleichen Chemotherapie während der RCT behandelt. Die Patienten wurden sechs Wochen nach der Radiochemotherapie operiert. Primärer Endpunkt war die Rate an vollständigen pathohistologischen Remissionen (pCR) bei Nonrespondern nach Wechsel der Chemotherapie.

## Ergebnisse

241 Patienten erhielten eine Behandlung gemäß Protokoll, von denen 225 Patienten eine auswertbare erneute PET-Bildgebung im Anschluss an die Chemotherapie erhielten. Die pCR-Rate für PET-Nonresponder nach Induktion mit FOLFOX und anschließendem Wechsel zu CP (*n* = 39) oder derer, die bei der Induktion mit CP zu FOLFOX wechselten (*n* = 50), betrug 18,0 % (95 %-KI 7,5–33,5) bzw. 20 % (95 %-KI 10–33,7). Die pCR-Rate bei Respondern, die eine FOLFOX-Induktionstherapie erhielten, betrug 40,3 % (95 %-KI 28,9–52,5) und bei den Patienten, die CP als Induktion erhielten, 14,1 % (95 %-KI 6,6–25,0). Das mediane Follow-up betrug 5,2 Jahre, die mediane Überlebenszeit 48,8 Monate für PET-Responder und 27,4 Monate für PET-Nonresponder. Für FOLFOX-Induktions-Patienten, die PET-Responder waren, wurde das mediane Überleben noch nicht erreicht.

## Schlussfolgerung der Autoren

Die frühzeitige Beurteilung des Therapieansprechens mit der PET-Bildgebung als Biomarker, um die Therapie für Patienten mit einem Adenokarzinom des Ösophagus oder ösophagogastralen Übergangs zu verbessern, ist effektiv und verbessert die pCR-Raten bei PET-Nonrespondern. PET-Responder auf die FOLFOX-Induktion und Fortführung von FOLFOX während der RCT erreichten ein vielversprechendes Gesamtüberleben (OS) nach 5 Jahren von 53 %.

## Kommentar

Der CALGB 80803 Trial steht im Kontext vieler aktueller Studien, die das Ziel verfolgen, das onkologische Outcome für Patienten mit lokal fortgeschrittenem Adenokarzinom des Ösophagus bzw. des ösophagogastralen Übergangs zu verbessern. Dies geschieht vor dem Hintergrund der deutlich zunehmenden Inzidenz dieser Entität in den westlichen Industrienationen – besonders im Vergleich zum Plattenepithelkarzinom [1]. Dabei steht neben einer perioperativen Chemotherapie eine neoadjuvante RCT mit anschließender Operation nach den aktuellen Leitlinien als primäre Therapieoption zur Wahl. Die optimale Kombinationstherapie ist zum aktuellen Zeitpunkt allerdings noch nicht festgelegt.

FDG-PET-CT-basierte Therapiekonzepte mit Anpassung der Therapie an die metabolische Tumoraktivität erfreuen sich immer größerer Aufmerksamkeit in der rezenten Literatur. Über signifikant schlechtere pCR-Raten von PET-Nonrespondern im Vergleich zu PET-Respondern unter Fortführung der Chemotherapie als präoperative RCT berichteten bereits verschiedene Studien [2, 3]. Konkret zeigten diese Studien bei den PET-Nonrespondern eine pCR-Rate von 4 bis 5 %. Die Idee der Autoren der hier kommentierten Arbeit war es, durch einen Wechsel der Chemotherapie bei PET-Nonrespondern die pCR-Rate als Surrogatparameter für das Therapieansprechen zu verbessern. Somit soll die FDG-PET-CT bzw. die metabolische Aktivität des Tumors unter der Therapie maßgeblich in das Therapiekonzept einbezogen werden. Konkret wurde mit dem Wechsel der Chemotherapie bei PET-Nonrespondern eine pCR-Rate von 20 % angestrebt. Die Ergebnisse der vorliegenden CALGB-80803-Studie zeigen nun, dass der primäre Endpunkt erreicht wurde: Bei PET-Nonrespondern kam es beim Wechsel der Chemotherapie von FOLFOX auf CP zu einer pCR-Rate von 18 %, beim Wechsel von CP auf FOLFOX zu einer pCR-Rate von 20 %. Gleichzeitig zeigte sich das mediane OS mit 27 Monaten in der Gruppe der PET-Nonresponder nicht signifikant kürzer als das der PET-Responder (49 Monate, *p* = 0,107). Somit konnte durch den Chemotherapiewechsel das Outcome für PET-Nonresponder näher an das der PET-Responder gebracht werden. Auffällig war zudem, dass PET-Responder, welche nur mit FOLFOX in der Phase der Induktion sowie in der Phase der neoadjuvanten Radiochemotherapie behandelt wurden, die beste pCR-Rate (40,3 %) sowie das beste 5‑Jahres-OS (53,0 %) hatten. Hieraus kann geschlussfolgert werden, dass FOLFOX als Induktionschemotherapie das beste Langzeitoutcome mit sich bringt, sodass zukünftige Studien sich auf dieses Induktionsschema konzentrieren sollten.

Um das onkologische Outcome des Therapiekonzepts der hier vorliegenden wissenschaftlichen Arbeit mit den aktuellen Therapiestandards der neoadjuvanten Radiochemotherapie bzw. der perioperativen Chemotherapie bewerten zu können, muss zunächst auf die Unterschiede der Studiendesigns eingegangen werden. Wichtig ist, dass im CALGB 80803 Trial die neoadjuvante Radiochemotherapie um eine 6‑wöchige Induktionschemotherapie mit FOLFOX/CP ergänzt wurde. Außerdem erhielten die Patienten eine Gesamtdosis von 50,4 Gy mit Einzeldosen von 1,8 Gy. Im Therapiestandard der neoadjuvanten RCT nach CROSS erhielten die Patienten hingegen nur 41,4 Gy in Einzeldosen von 1,8 Gy [4, 5]. Das Chemotherapieschema war hingegen identisch zur simultanen CP-Phase von CALGB 80803 mit Carboplatin AUC 2 sowie Paclitaxel 50 mg/m^2^ „weekly“ für fünf Wochen. Hinsichtlich des onkologischen Outcomes zeigte sich das OS nach 5 Jahren sowie die pCR-Rate der nach CROSS behandelten Patienten vergleichbar (OS bei CROSS 43 % vs. CALGB 80803 45 %; pCR-Rate 23 % bei CROSS vs. 24,4 % bei CALGB 80803). Mit 37 % Grad-III–IV-Toxizitäten in der vorliegenden Studie zeigten die Patienten etwas mehr Toxizität als nach CROSS (8 %/11 %).

Im aktuellen Therapiestandard der perioperativen Chemotherapie nach FLOT4 kam hingegen eine Chemotherapie mit vier prä- sowie vier postoperativen Zwei-Wochen-Zyklen FLOT mit 50 mg/m^2^ Docetaxel, 85 mg/m^2^ Oxaliplatin, 200 mg/m^2^ Leucovorin sowie 2600 mg/m^2^ 5‑FU als 24-h-Infusion zur Anwendung [6]. Auch hier zeigt sich das onkologische Outcome vergleichbar zwischen FLOT4 und CALGB 80803 (FLOT4 5‑Jahres-OS 39 %, pCR-Rate 16 %). Im Gegensatz zur neoadjuvanten Chemotherapie scheint hier allerdings die Toxizität im Vergleich mit der vorliegenden Studie etwas ausgeprägter zu sein (51 % Grad-III–IV-Toxizitäten).

Insgesamt zeigt der vorliegende CALGB 80803 Trial einige Stärken, aber auch Limitationen, die bei der Interpretation der Ergebnisse berücksichtigt werden müssen. Eine liegt darin, dass formal keine PET-basierte Randomisierung erfolgte, wodurch der wirkliche Effekt noch genauer hätte untersucht werden müssen. Weiterhin ist eine Induktionschemotherapie beim lokal fortgeschrittenen Adenokarzinom des Ösophagus bzw. des ösophagogastralen Übergangs aktuell kein etablierter Standard. Trotzdem muss hervorgehoben werden, dass die Studie über ein sehr aufwendiges Studiendesign verfügt und einen Schritt weiter geht in Richtung Personalisierung der onkologischen Therapie. Es konnten immerhin auch 225 Patienten, speziell mit Adenokarzinomen, eingeschlossen werden, das Follow-up war zufriedenstellend mit mehr als fünf Jahren Nachbeobachtung.

Studien zur Therapieoptimierung beim lokal fortgeschrittenen Adenokarzinom des Ösophagus bzw. des AEG-Tumors stellen wichtige und interessante Therapieansätze in der Zukunft dar. Aktuell sind folgende zu nennen: Die ESOPEC-Studie ist eine Phase-III-Studie, welche die Effizienz der neoadjuvanten Radiochemotherapie nach CROSS mit der perioperativen Chemotherapie nach FLOT4 vergleicht. Finales Rekrutierungsziel ist das Jahr 2024 [7]. Derselben Fragestellung geht die Neo-AEGIS-Studie nach, deren Zwischenanalyse dieses Jahr Hinweise auf eine Nichtunterlegenheit der perioperativen Chemotherapie gegenüber der neoadjuvanten Radiochemotherapie zeigte [8]. Die endgültigen Ergebnisse bleiben abzuwarten.

## Fazit

Der CALGB 80803-Trial als Phase-II-Studie erreicht bei Patienten mit Adenokarzinomen des Ösophagus sowie mit AEG-Tumoren eine potenzielle Verbesserung der pCR-Raten sowie ein gutes onkologisches Outcome durch ein PET-CT-basiertes Response-Assessment der neoadjuvanten Radiochemotherapie nach initialer Induktionschemotherapie. Auf Basis der hier besprochenen Daten könnten weitere prospektive Studien zu personalisierten Therapiealgorithmen ansetzen, die die metabolischen Aktivitäten des Tumors mit in die Behandlung einbeziehen. Die aktuellen Phase-III-Studien ESOPEC und Neo-AEGIS werden sicherlich weitere interessante Aspekte eröffnen.

*Lukas Bauer und Kristin Lang, Heidelberg*
